# Double-Edge, Single-Edge, and Intermediate-Edge Ultrasound Sign and Correlation With Fascial Plane Block Efficacy: An Experimental Study

**DOI:** 10.7759/cureus.51858

**Published:** 2024-01-08

**Authors:** Vasileios Boviatsis, Alexios Triantopoulos

**Affiliations:** 1 Anesthesiology, General Hospital of Patras, Patras, GRC

**Keywords:** methylene blue, ultrasonography, analgesia, regional anesthesia, cadaver, fascia, peripheral nerves

## Abstract

Introduction

Ultrasound-guided fascial plane blocks are nowadays the gold standard technique for regional anesthesia and postoperative analgesia. Despite their high success rate, cases of partial or total failure of this method have been reported. This experimental study aims to address the corresponding ultrasound signs and their association with fascial plane block efficacy.

Methods

After capturing the appropriate sonographic image that included muscle layers and their fasciae, an 18-gauge epidural needle penetrated the cadaveric porcine tissue and was forwarded until the tip of the needle reached the target fascial plane. The infusion of methylthioninium chloride or methylene blue dye was performed, causing tissue hydro dissection. The documentation of the generated ultrasound images was followed by surgical exposure of the tip of the needle.

Results

The distribution of the dye into the plane of interest (double-edge sign) was equivalent to block success, whereas the single-edge sign (accumulation of the dye between fascia and epimysium) indicated total block failure. The intermediate-edge sign, a combination of the previous ultrasound signs, is related to partial failure of block performance.

Conclusion

The identification of the three novel sonographic signs is an accurate predictive factor of peripheral nerve block efficacy. The respective data are expected to aid the rapid improvement of interfascial plane block accuracy and techniques, leading to their more effective execution and simultaneously eliminating the failure rates. Thereby, the amelioration of intra and postoperative analgesia will be accomplished, expediting the patient's hospital discharge and reducing or even avoiding opioid consumption.

## Introduction

Regional anesthesia techniques are widely used nowadays, providing not only primary anesthesia in a variety of surgeries but also efficient postoperative analgesia, shorter hospitalization time, and lower doses or complete avoidance of opioid consumption [1,2]. The landmark-guided technique has been replaced today by the use of ultrasound for forwarding the tip of the needle into the space between the two fascial layers, which contain the anatomical course of nerves, and injecting the local anesthetic under direct visualization, without affecting nerves' integrity [3,4]. As a result, a higher success rate and lower incidence of injury of the nerves and adjacent anatomic structures have been accomplished when performing a peripheral nerve block [1,3,4].

Despite the popularity of fascial plane blocks, cases of partial or total failure of this method have been described, according to the literature [5,6]. Poor skills of the operator, multiple ways of possible access to the target nerves, and factors that affect the ultrasound visibility, like excessive subcutaneous adipose tissue, lead to both inappropriate pain relief and the necessity for rescue analgesia postoperatively [5-7]. However, there are no studies that document ultrasound images, depicting the plane of the needle’s tip placement, followed by local anesthetic injection, and relating this evidence to potential success or failure of peripheral nerve block. Hence, an experimental study on cadaveric porcine tissues was conducted, aiming to highlight the above ultrasound signs and their correlation with fascial plane block efficacy and provide significant information for the filling of this particular knowledge gap in the literature.

A part of this article was previously presented as a meeting abstract at the 21^st^ Panhellenic Congress on Regional Anesthesia, Pain Management, and Palliative Care on September 16-19, 2021.

## Materials and methods

The experimental study was performed by the Department of Anesthesiology of the General Hospital of Patras from January 2021 to August 2023 and was approved by the Scientific Committee of the General Hospital of Patras. Taking into consideration the anatomic similarities between human and porcine abdominal wall structures, the conduction of the experiments took place on cadaveric pig models [8]. Except for the abdominal wall, any other parts of the pig cadaveric body were excluded from the study. The usage of pig cadaver material incurred from a non-related non-survival study. The goal was to simulate the needle’s tip positioning and local anesthetic spread within the fascial plane, where the target nerves are located, during the performance of the fascial plane block under ultrasound guidance. The following steps of the execution of the simulated fascial plane block were performed by an experienced anesthesiologist with the assistance of a trainee resident of anesthesiology.

Technique

To begin with, a linear ultrasound probe was necessary for the identification of the framework of the abdominal wall [7]. The puncture on the cadaveric specimen was made after the particular sonographic image included muscle layers and their fasciae [7]. An 18-gauge epidural needle penetrated the abdominal wall and was forwarded until it reached the fascial space of interest. The needle’s size was decided, according to the recommendations for improving the needle’s visibility [9]. Then, infusion of a dye, methylthioninium chloride or methylene blue, was performed through the epidural needle, instead of local anesthetic, causing tissue hydro dissection. The documentation of the generated ultrasound images was followed by surgical exposure of the tip of the needle. In some cases, a second 18-gauge epidural needle was placed at the plane of the injected dye, guided by sonography, and used as a guide needle for establishing surgical access to the tip of the first needle.

Outcome measures

The ultrasound images, after the distribution of the dye and the revelation of the epidural needle’s tip, during subsequent surgical dissection of the cadaveric tissues, were registered. Afterward, their impact on the fascial plane block’s clinical outcome was evaluated. The accumulation of the blue dye into the target plane was defined as the successful execution of the interfacial plane block while any other possible spread pattern was equivalent to partial or total failure of the block.

## Results

In terms of our study, three sonographic signs were obtained and their influence on the efficacy of peripheral nerve block is summarized in Table 1.

**Table 1:** Ultrasound signs and their corresponding results on block efficacy

Ultrasound sign	Result
Double–edge sign	Success
Single-edge sign	Failure
Intermediate-edge sign	Partial failure

Double-edge sign

**Figure 1:**
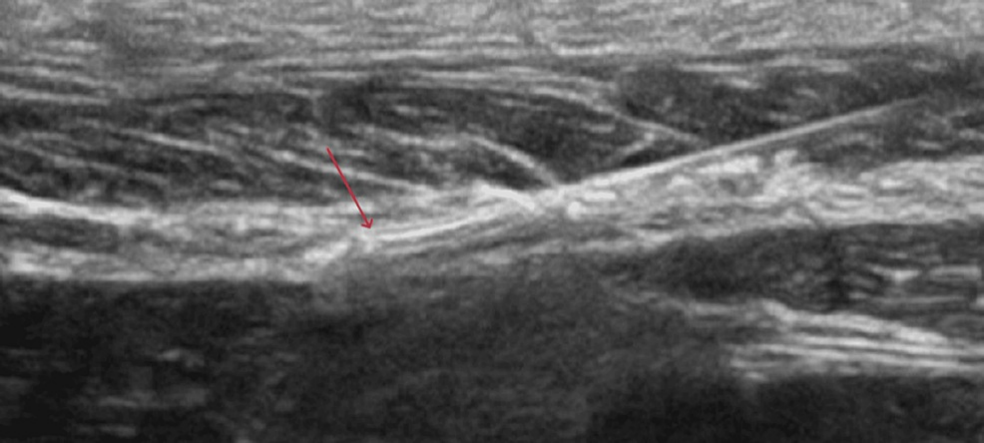
The tip of the needle (red arrow)

**Figure 2:**
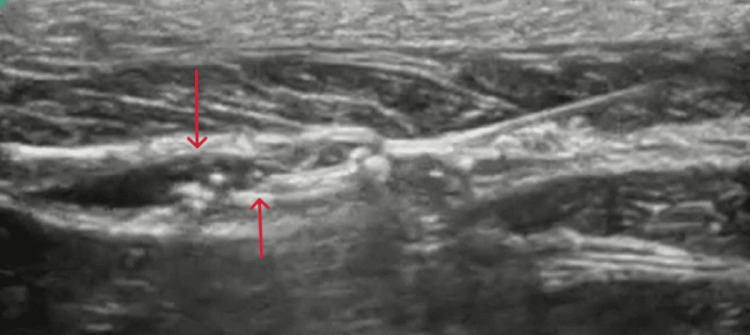
The dark spindle-like shape of the distribution of the dye between the fascial layers (red arrows)

**Figure 3:**
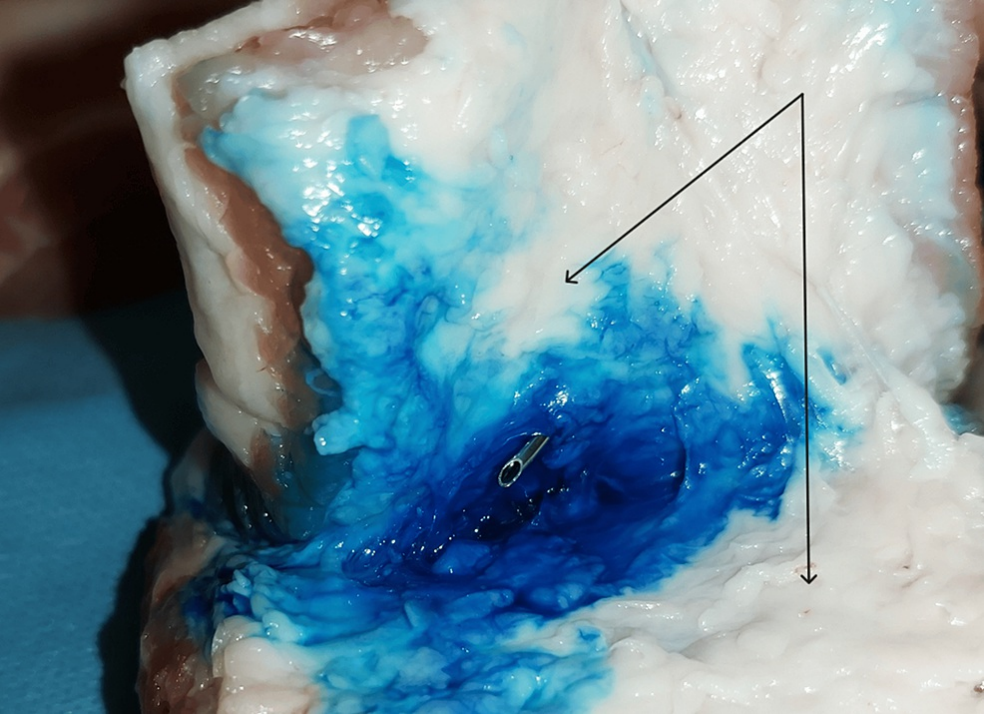
The surgical exposure of the tip of the needle (black arrows): fascial layers

The double-edge sign referred to the distribution of the dye into the requested fascial plane after the right needle’s placement via ultrasound guidance (Figure 1). It indicated the successful hydro dissection of the two fasciae, creating a dark spindle-shaped area (Figure 2). The two clearly separated hyperechogenic fasciae of the target fascial plane that surrounded the precisely delineated hypoechogenic area of the blue dye’s spread into the interfascial space were defined as a double-edge sign, during the acquisition of the ultrasound image (Figure 2). The subsequent surgical exposure confirmed the accumulation of the blue dye between the two fascial layers (Figure 3).

Single-edge sign

**Figure 4:**
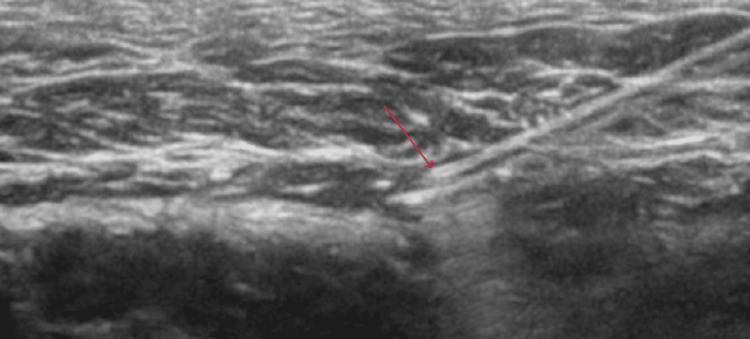
Incorrect placement of the tip of the needle (red arrow)

**Figure 5:**
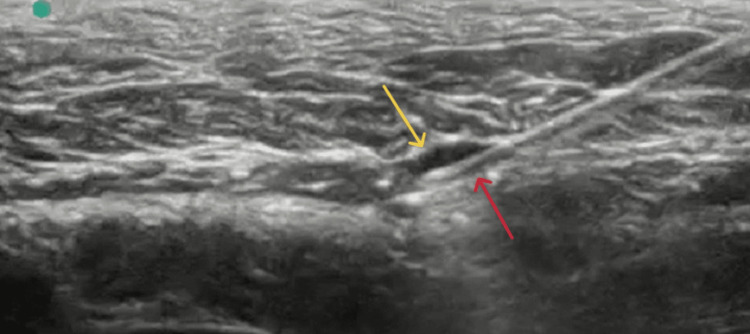
Injection of the blue dye and formation of the almost spindle-shaped hypoechogenic area between the epimysium (yellow arrow) and one fascial layer (red arrow)

**Figure 6:**
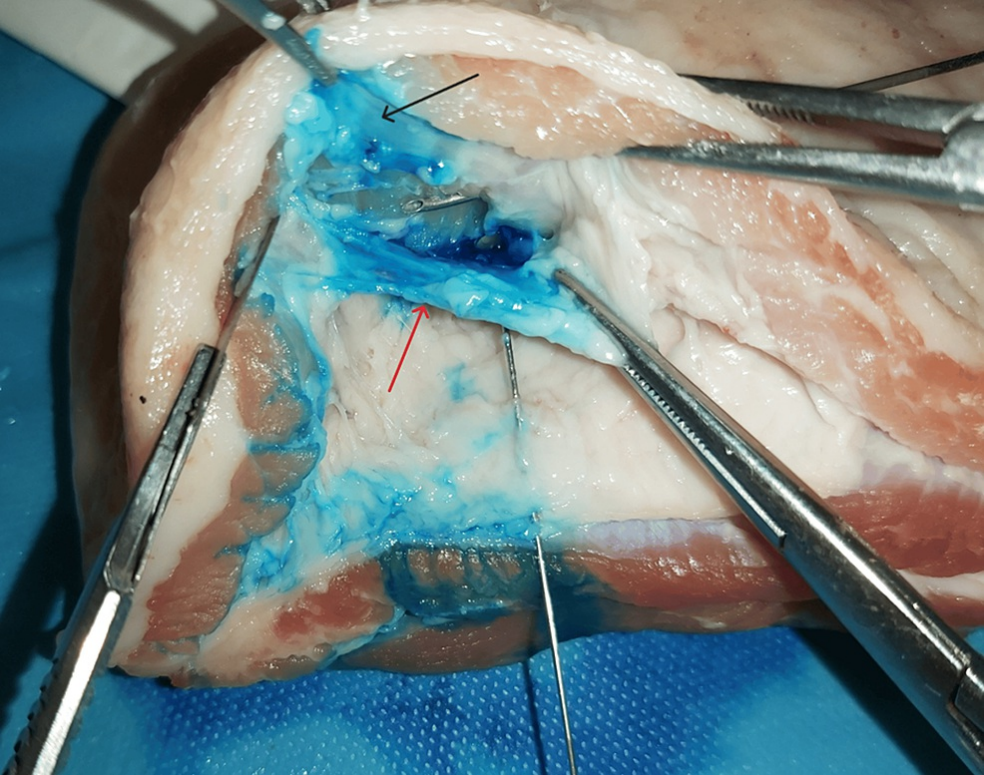
Accumulation of the blue dye between epimysium (black arrow) and fascia (red arrow)

**Figure 7:**
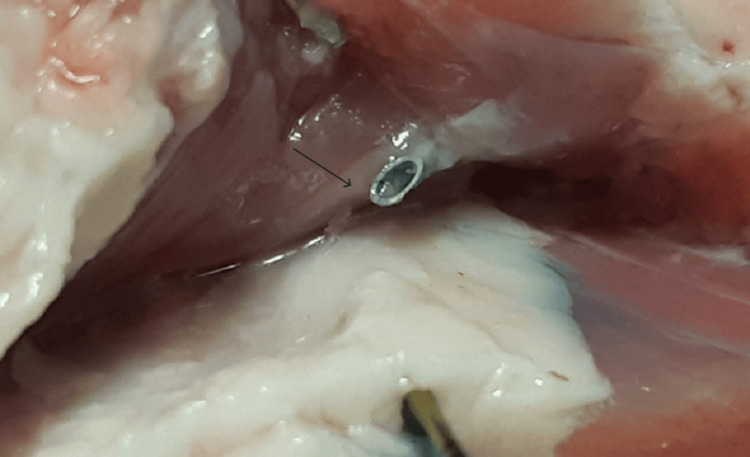
Exposure of epimysium (black arrow)

On the other hand, the single-edge sign was equivalent to failure of the peripheral nerve block. Specifically, the tip of the needle was thought to be positioned at the right fascial space (Figure 4) and the ultrasound image captured an almost-formed spindle-shaped hypoechogenic area, as explained before (Figure 5). However, the following surgical dissection of the cadaveric sample revealed that the tip of the needle had not penetrated the fascia, as expected, and the blue dye was injected between the fascia and epimysium, fibrous tissue that normally surrounds skeletal muscle (Figures 6, 7). After the injection of the dye, the sonographic image depicted the formation of an almost spindle-shaped hypoechogenic area, which was endued by two hyperechogenic structures, the one fascial layer of the target fascial plane and the epimysium. The particular ultrasound image was determined as a single-edge sign because it contained only one of the two fasciae of the requested fascial plane (Figure 5).

Intermediate-edge sign

**Figure 8:**
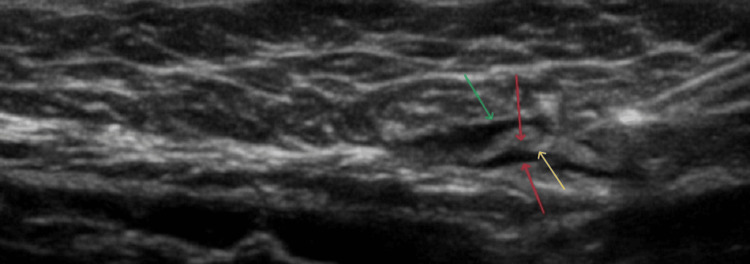
Red arrows: fascial layers, yellow arrow: tip of the needle, green arrow: epimysium

**Figure 9:**
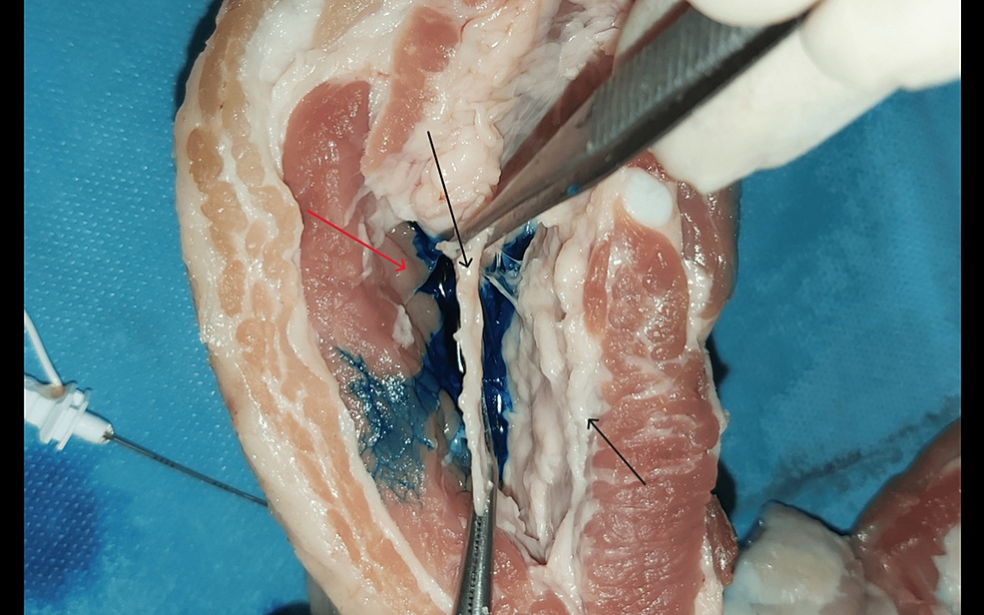
Black arrows: fascia layers, red arrow: epimysium

**Figure 10:**
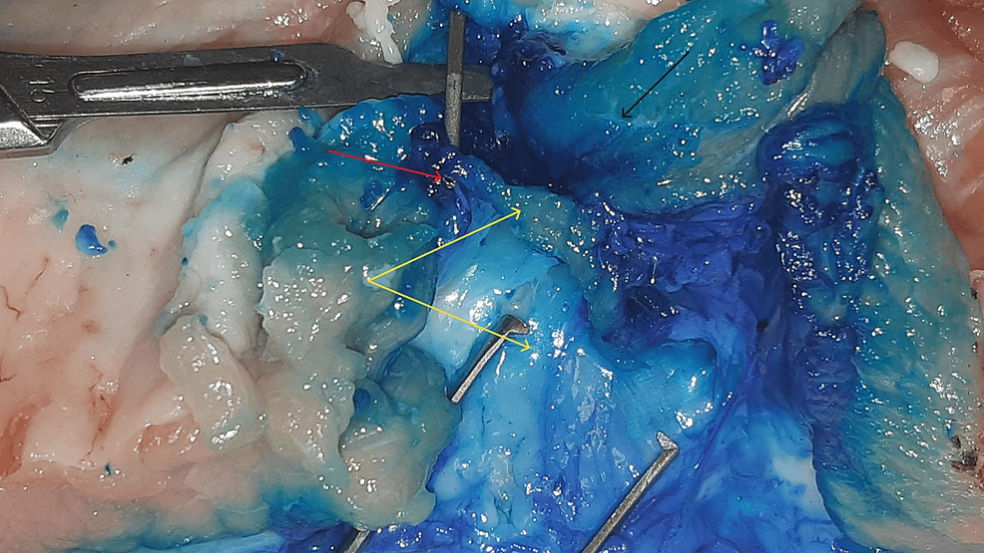
Yellow arrows: fasciae, red arrow: tip of the needle, black arrow: epimysium

**Figure 11:**
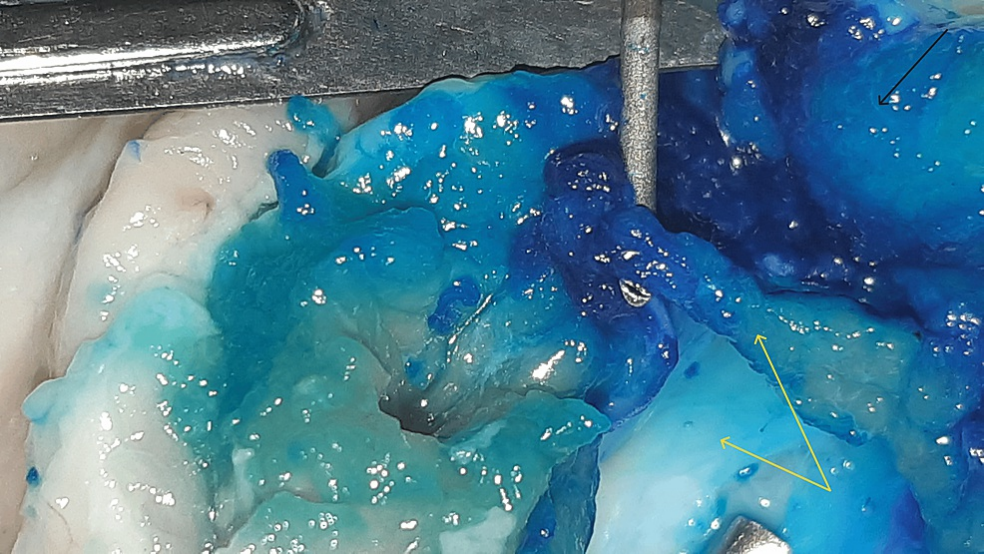
Enlargement of Figure 10 with a distinct vision of the tip of the needle, fasciae (yellow arrows), and epimysium (black arrow)

The third sonographic mark was a combination of the previous findings. According to the obtained ultrasound image (Figure 8), the separation of the adjacent tissues, after the infusion of the dye, was accompanied by the display of an obvious hyperechogenic structure inside the created hypoechogenic area. When surgically exposed, the depicted structure belonged to one layer of the target fascial plane, which implied that the distribution of the blue dye took place, not only between the requested fascial layers but also between fascia and epimysium (Figures 9-11). Consequently, the respective sonographic sign was defined as an intermediate-edge sign, as the one fascial layer (intermediate hyperechogenic structure) of the requested fascial plane divided the formed hypoechogenic area (distribution of the dye), during ultrasound imaging, which was enclosed by two hyperechogenic structures, the second fascial layer and the epimysium correspondingly (Figure 8). This evidence may advocate the partial failure of the peripheral nerve block.

## Discussion

In this experimental study, we report the initial data, derived from the injection of blue dye between cadaveric porcine tissue layers, aiming to simulate the implementation of an interfascial plane block on a patient, requiring either regional anesthesia or postoperative analgesia. Specifically, after the ultrasound-guided localization of the adequate fascial plane, the infusion of the blue dye was performed, via an epidural needle, causing tissue hydro dissection. Its dispersion was identified through subsequent surgical exposure. Three sonographic images were accomplished and their relation to peripheral nerve block efficacy was interpreted.

The formation of a hypoechogenic spindle-shaped area, after recognition of the separated fascial layers during sonographic imaging, was defined as a double-edge sign, due to the two hyperechogenic fasciae, surrounding the target plane. The surgical verification of the blue dye’s spread indicated the successful result of the interfascial plane block. A similar ultrasound sign, named double-V sign, was recently described and it was associated with the correct application of the block technique and achievement of adequate analgesia [10]. These findings underline the clinical importance of the incorporation of ultrasound into regional anesthesia and analgesia techniques since it both facilitates the execution of peripheral nerve block and eliminates the possibility of complications with a simultaneous increase in success rate [1,11,12]. The real-time imaging makes feasible the correct deposition of local anesthetic proximal to the route of nerves, that travel into the fascial compartment, which is full of a fat-glycosaminoglycan matrix, through a bulk flow [13,14].

Nevertheless, the complete success of interfascial plane blocks and management of postoperative pain remains challenging, although the evolution of technological equipment during the last years has been noted [1,5,6,15]. Factors affecting block efficacy, such as decreased ultrasound visibility, due to obesity, and variation, in methods of approaching the plane of interest, have been clearly pointed out [7,11,12]. We should not ignore that peripheral nerve blocks offer only parietal analgesia, without the ability to control visceral pain, and, of course, the errors, concerning the operator’s technique and site of injection of local anesthetic, as mentioned by many authors [5,6,12].

In the current study, two novel sonographic images that are compatible with undesirable block failure are demonstrated. During the formation of the single-edge sign, the blue dye was distributed outside the target plane, between the fascia and epimysium. On the contrary, the accumulation of the blue dye into two spaces, between the two fasciae and between the fascia and epimysium, divided by the hyperechogenic fascia layer of the plane of interest, led to the corresponding ultrasound image of an intermediate-edge sign. Except for the surgical verification of the above findings, the understanding of these ultrasound signs and their negative prediction for block efficacy necessitates the comprehension of dispersion patterns of local anesthetics and fascial framework [14,16].

The spread of local anesthetic follows mainly the pattern of bulk flow into the fascial plane, which is the pathway for nerves and vessels [14,17]. In addition, the fluid can be diffused into surrounding tissues, through pores of the permeable fascial layers, even if no perforation has occurred, affecting local nociceptors [14,17]. The above mechanisms of medication distribution may explain the partial failure of peripheral nerve block when accompanied by the ultrasound image of the intermediate-edge sign, as only a part of the administered local anesthetic’s volume can reach the nerves inside the target fascial plane. Contrarily, the infusion of local anesthetic between the fascia and high-density tissue of epimysium, which corresponds to the single-edge sign, indicates total block failure because the main volume of liquid is injected distally to the target plane, with unknown clinical impact, caused by intramuscular dispersion [16,18]. Furthermore, we should bear in mind the differences between the fasciae of the human body, regarding their location, anatomy, mobility, and adjacency with organs and tissues and how these factors influence local anesthetic’s spread, which requires more investigation [16]. Either targeting any fascial compartment or depositing local anesthetic on the same fascial plane by a different approach does not guarantee that the expected sensory and analgesic blockade will be accomplished [11,16,19]. Finally, great care should be taken of the administered volume of local anesthetics to avoid complications from toxic plasma concentrations, as they can be absorbed by systemic circulation after their injection [14,17,19].

Taking everything into account, it is essential to acknowledge explicitly the limitations of this study. The main limitations of our study are its experimental nature and its conduction on pig cadavers. The relevance of the above novel ultrasound signs to the efficacy of peripheral nerve blocks should be further examined, through the design of future studies, where these sonographic signs will be generated and evaluated using living animal models and human participants. Moreover, the fact that the experimental material consisted only of porcine abdominal walls is another constraint of the present study. Notwithstanding the anatomical resemblances of the human and porcine abdominal wall frameworks, the variations in the human body's fascial structures, encompassing their position, anatomy, mobility, and proximity to organs and tissues, and how these elements impact the spread of local anesthetics necessitate upcoming research endeavors on alternative living or cadaveric human tissues, which will focus on the microanatomy of the target fascial plane and the dispersion pattern of local anesthetic injection This approach will provide a more comprehensive understanding of the practical implications and potential clinical benefits of the identified ultrasound signs in the context of peripheral nerve block efficacy.

## Conclusions

Ultrasound-guided fascial plane blocks are the primary option for regional anesthesia and postoperative analgesia. The prediction of block efficacy is attributed to the recognition of specific ultrasound signs. The accumulation of local anesthetic into the target fascial plane (double-edge sign) is associated with block success while the infusion of local anesthetic between the fascia and epimysium indicates total failure of the block (single-edge sign). Finally, the intermediate-edge sign, a combination of both previous sonographic signs, is indicative of the partial failure of block performance. The respective data are expected to aid the rapid improvement of interfascial plane block accuracy and techniques, leading to their more effective execution and simultaneously minimizing the failure rates. Thereby, the amelioration of intra and postoperative analgesia will be accomplished, expediting the patient's hospital discharge and reducing or even avoiding opioid consumption.
